# Identification of cutaneous fungi and mites in adult atopic dermatitis: analysis by targeted 18S rRNA amplicon sequencing

**DOI:** 10.1186/s12866-021-02139-9

**Published:** 2021-03-04

**Authors:** Sofie Marie Edslev, Paal Skytt Andersen, Tove Agner, Ditte Marie Lindhardt Saunte, Anna Cäcilia Ingham, Thor Bech Johannesen, Maja-Lisa Clausen

**Affiliations:** 1 Department of Bacteria, Parasites, and Fungi, Statens Serum Institut, Copenhagen, Denmark; 2 Department of Veterinary and Animal Sciences, University of Copenhage, Frederiksberg, Denmark; 3 Department of Dermatology, Bispebjerg University Hospital, Copenhagen, Denmark; 4 Department of Dermatology, Zealand University Hospital, Roskilde, Denmark; 5 Department of Clinical Medicine, University of Copenhagen, Copenhagen, Denmark

**Keywords:** Atopic dermatitis, Microbiome, Fungi, Mycobiome, Demodex, Malassezia

## Abstract

**Background:**

Atopic dermatitis (AD) patients have an altered skin bacterial community, with an abundance of *Staphylococcus aureus* associated with flares, highlighting that microbial organisms may be important for disease exacerbation. Despite strong evidence of association between bacterial skin colonisation and AD, very limited knowledge regarding the eukaryotic microbial community, including fungi and ectoparasites, in AD exists. In this study, we compared the skin and nasal eukaryotic microbial community between adult AD patients (*n* = 55) and non-AD healthy controls (*n* = 45) using targeted *18S rRNA* amplicon sequencing. Analysis was based on the presence or absence of eukaryotic microorganisms.

**Results:**

The cutaneous composition of the eukaryotic microbial community and the alpha-diversity differed significantly between AD patients and non-AD individuals, with increased species richness on AD skin. Alpha-diversity and beta-diversity were similar on lesional and non-lesional skin of patients. The ectoparasite *Demodex folliculorum* and the yeast *Geotrichum candidum* were significantly more prevalent on the skin of AD patients. The prevalence of *D. folliculorum* on lesional skin was greater among patients recently treated with topical corticosteroid. *Malassezia* was one of the most frequently detected genera at all sites, with *M. globosa* and *M. restricta* being the most prevalent. *M. restricta* was under represented in the anterior nares of AD patients as compared to the non-AD control population.

**Conclusion:**

Significant differences in the eukaryotic microbial communities were found between AD patients and non-AD individuals, with the most striking finding being the significantly overrepresentation of *D. folliculorum* on AD skin. Whether *D. folliculorum* can contribute to skin inflammation in AD needs further investigation.

**Supplementary Information:**

The online version contains supplementary material available at 10.1186/s12866-021-02139-9.

## Background

Atopic dermatitis (AD) is a common inflammatory skin disease, affecting up to 20% of children and 3–5% of adults in developed countries [1, 2]. The disease is complex and multifactorial and includes impaired skin barrier function and altered immune response [3]. Red, dry, itchy skin characterizes the disease, with repeated flares linked to disturbances in the skin microbial environment [4].

The skin microbiota is a complex community consisting of diverse organisms including bacteria, fungi, and parasites. It is well known that *Staphylococcus aureus* skin colonisation is common among AD patients, associated with decreased bacterial diversity and increased disease severity [4–7]. While several studies have examined the bacterial community on skin, our knowledge regarding the eukaryotic microbial community on AD skin is very limited. Two sequencing-based studies have shown that the fungal richness and diversity are greater on AD lesional skin (LS) compared to healthy control skin [8, 9]. *Malassezia* species, especially *M. globosa* and *M. restricta,* are the most common and abundant fungal species on skin of AD patients and healthy individuals [8–12]. *Malassezia* has been implicated in AD pathogenesis, as patients are more often hypersensitive with specific IgE antibodies against *Malassezia* in comparison to healthy individuals, a subtype of AD called ‘head and neck dermatitis’ [13–15]. *Candida* is another commensal yeast suggested to contribute to the onset and exacerbation of AD [14], but a higher colonisation rate of AD patients compared with controls has not been found [16]. Furthermore, a wide range of environmental molds, such as *Aspergillus* and *Cladosporum* species, can induce type I allergic responses, where greater incidence rates have been reported among atopic patients compared to the general population [17].

In this explorative study, we aimed to compare the presence of skin colonising fungi and parasites on adult AD patients and non-AD healthy individuals, using *18S rRNA* gene amplicon sequencing. Furthermore, we aimed to investigate whether topical corticosteroid (TCS) treatment, AD disease severity and AD risk factors (filaggrin gene (*FLG*) mutations and *S. aureus* colonisation) were associated with changes in the eukaryotic microbial community.

## Results

Samples were collected from skin and the nares of 58 adult AD patients and 46 non-AD healthy individuals, including both lesional (LS) and non-lesional (NLS) skin areas from the AD patients. Three patients were excluded prior to analysis due to low quality of samples, and 55 AD patients were thus included in the final analysis. Demographic and clinical descriptions of the study population are given in Table 1.

**Table 1:** Characteristics of study participants

Characteristics		Atopic dermatitis patients (***n*** = 55)	Non-AD healthy individuals (***n*** = 46)
Gender	Female	29 (53%)	26 (57%)
	Male	26 (47%)	20 (43%)
Age (years)	Median (range)	35 (18–77)	42 (26–69)
SCORAD^a^	Mean	30.9	–
	Mild:Moderate:Severe	18:31:6	–
*FLG* status	WT	31 (56%)	–
	Mutations	19 (35%)	–
	Unknown	5 (9%)	–
*S. aureus colonization*	Culture positive in LS	29 (53%)	–
	Culture positive in NLS	15 (27%)	–
	Culture positive in nose	30 (55%)	–
Atopy	Asthma	17 (31%)	–
	Hayfever	36 (66%)	–
	Allergy	39 (71%)	–
Treatments	Topical corticosteroid^b^	42 (76%)	1 (2%)
	Topical calcineurin inhibitor^c^	13 (24%)	1 (2%)
	Antibiotics (total)^d^	16 (29%)	3 (7%)
	Systemic antibiotics^d^	11 (20%)	3 (7%)
	Topical antibiotics^d^	8 (15%)	0
	Systemic treatment for AD^e^	18 (33%)	0
Skin type of sample location^f^	LS (Dry:Moist:Sebaceous)	23:8:11	–
	NLS (Dry:Moist:Sebaceous)	8:45:1	0:46:0

a: SCORAD groups were defined as: Mild AD (SCORAD< 25), Moderate AD (SCORAD 25–50), and Severe AD (SCORAD> 50). b: Treatment within the last month prior to sampling timepoint. The majority of AD patients were treated within one week before sampling (*n* = 38). All corticosteroids were of group III. c: Treatment within the last three months. Topical calcineurin inhibitors (tacrolimus or pimecrolimus) had only been applied in the face, and no skin samples had been collected from facial areas. d: Treatment within the last three months prior to sampling. 6/8 patients treated with topical antibiotics and 4/11 patients treated with oral antibiotics had been treated within one week prior to sample collection. e) Treatment within the last three months prior to sampling. Systemic treatment for AD includes Methotrexate (*n* = 6), azathioprin (*n* = 7), prednisolone (*n* = 3) and alitretionine (*n* = 3). f: Missing information on LS skin location for 13 patients and missing information on NLS skin location for one patient. *Abbreviations*: *AD* atopic dermatitis, *LS* lesional skin, *NLS* non-lesional skin, *SCORAD* Severity Scoring of Atopic Dermatitis, *FLG* filaggrin gene, *WT* wildtype

Compositional analysis of the eukaryotic microbial communities on skin and in nares was based on the presence or absence of specific organisms, where species presence was defined as ≥10 classified sequence reads within a sample (See the Methods Section for more details).

AD skin samples were collected from distinct anatomical sites depending on the presence of eczema, with LS samples primarily collected at dry skin areas (*n* = 23) such as the volar forearm and dorsal hand, and NLS samples were primarily collected from moist skin areas of the antecubital crease (*n* = 45) (Table 1). All skin samples from healthy individuals were also collected from the antecubital crease. Initial analysis indicated that differences in the microenvironment of the sampling area (i.e. dry, moist or sebaceous skin) did not significantly influence the overall eukaryotic microbial composition on either LS or NLS (Additional file 1: Fig. S2) and differences in anatomical sites being sampled between individuals were therefore considered not to significantly influence subsequent analyses.

### Eukaryotic microbial community composition and richness

**Fig. 1:**
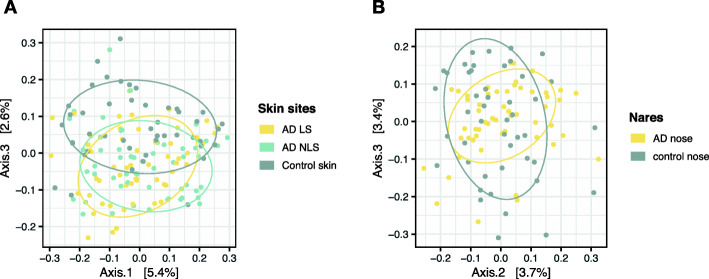
Eukaryotic microbial community composition across sample sites. Differences in the eukaryotic microbial community composition on skin (**a**) and in anterior nares (**b**) between AD patients and non-AD healthy controls were examined using Jaccard distances and here visualized using principal coordinates analysis (PCoA). The analysis of similarity test (ANOSIM) was used for pairwise comparison between the following sample sites: i) AD LS and NLS (*R* = 0.00, *p* = 0.48), ii) AD LS and control skin (*R* = 0.12, *p* = 0.0001, adjusted *p* = 0.0012), iii) AD NLS and control skin (*R* = 0.10, *p* = 0.0001, adjusted *p* = 0.0012) and iv) AD nose and control nose (*R* = 0.06, *p* = 0.003, adjusted *p* = 0.03). *P*-values were adjusted for mass significance using the Bonferroni method. Abbreviations: AD: atopic dermatitis, LS: lesional skin, NLS: non-lesional skin

The eukaryotic microbial community composition on AD skin was significantly different from the composition on healthy control skin, for both AD LS (*R* = 0.12, adjusted *p* = 0.001; ANOSIM) and AD NLS (*R* = 0.10; adjusted *p* = 0.001; ANOSIM), though the close clustering of some AD and control skin samples indicates some degree of similarity between sites (Fig. 1a). No differences were observed between AD LS and NLS (*R* = 0.00; ANOSIM).

**Fig. 2:**
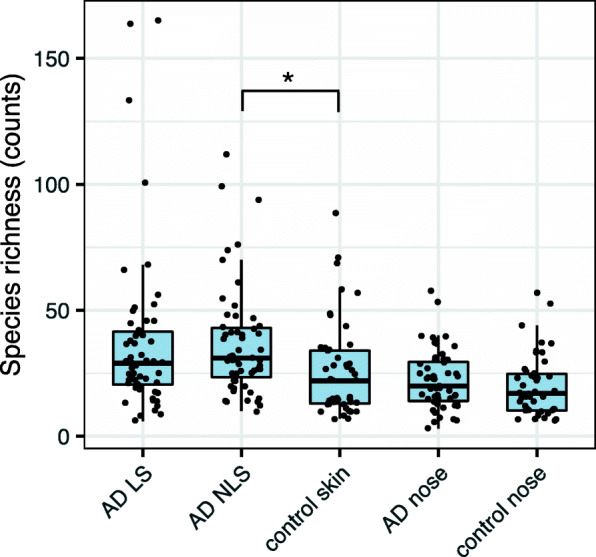
Eukaryotic microbial species richness across sample sites. Differences in species richness, were tested between the following sample groups: i) AD LS and NLS (*p* = 0.6), ii) AD LS and non-AD healthy control skin (*p* = 0.02, adjusted *p* = 0.3), iii) AD NLS and healthy control skin (*p* = 0.002, adjusted *p* = 0.02), and iv) AD nose and healthy control nose (*p* = 0.2). A Mann-Whitney U test was performed for paired samples and Wilcoxon Signed Rank test for unpaired samples. * marks a statistically significant difference between compared sample sites (Bonferroni adjusted *p*-value < 0.05). Boxes correspond to the 1. quartile, median, and 3. quartile. Whiskers extend to samples with the minimum/maximum count, but no longer than 1.5 x IQR (Inter-Quartile-Range). Dots represent individual samples. Abbreviations: AD: atopic dermatitis, LS: lesional skin, NLS: non-lesional skin

The eukaryotic microbial richness, defined as the total number of observed species in a sample, was overall higher on skin compared to the anterior nares (Fig. 2). Richness was greater on both AD LS (median species count: 29.0 (IQR: 20.5–41.5)) and AD NLS (31.0 (23.5–43.0)) compared to healthy control skin (22.0 (13.0–34.0)), though after correcting for multiple testing only statistically significant between AD NLS and healthy control skin (AD LS vs control skin: *p* = 0.03, adjusted *p* = 0.3; AD NLS vs. control skin: *p* = 0.002, adjusted *p* = 0.02, Mann-Whitney U test). No significant difference in richness was observed between AD LS and NLS.

Next, we examined whether TCS treatment within one month prior to sampling could influence the diversity on AD LS, and there we observed a significant variation in community composition between the treated (*n* = 42) and non-treated patients (*n* = 13) (*R* = 0.20, *p* = 0.006, adjusted *p* = 0.07; ANOSIM) (Additional file 1: Fig. S3). Also, the species richness on AD LS was greater among patients who had been treated with TCS (30.0 (22.5–45.8)) compared to non-treated patients (20.0 (14.0–32.0)), but the difference was not statistically significant after correcting for multiple testing (*p* = 0.046, adjusted *p* = 0.4, Mann-Whitney U test) (Additional file 1: Fig. S3).

Examination of the eukaryotic microbial community in the nares, showed a statistically significant, but minor difference in community composition between AD patients and healthy controls (*R* = 0.06, adjusted *p* = 0.03; ANOSIM) (Fig. 1b). The species richness in the anterior nares did not differ between AD patients (20.0 (14.0–29.5)) and healthy controls (17.0 (10.3–24.8)) (Fig. 2).

### Single species overrepresentation on atopic dermatitis skin

Two species, the skin mite *Demodex folliculorum* and the yeast *Geotrichum candidum,* were significantly more common on AD skin compared to healthy control skin (Table 2) (Additional file 1: Fig. S4 and Fig. S5). *D. folliculorum* was present in 69% of AD NLS and 60% of AD LS, which was significantly more than the 15% presence on healthy control skin (AD LS vs control skin: adjusted *p* <  0.003; AD NLS vs control skin: adjusted *p* <  0.0001, Fisher’s exact test). Furthermore, the prevalence of *D. folliculorum* on AD LS was greater among patients treated with TCS within 1 month prior to sample collection (OR: 4.9 (95% CI: 1.1–25.7), *p* = 0.02, adjusted *p* = 0.07, Fisher’s exact test).* G. candidum* was significantly overrepresented on AD LS (36% presence) compared to healthy control skin (4%) (adjusted *p* = 0.04, Fisher’s exact test). The same trend was observed for AD NLS (35% presence) (*p* <  0.0002, adjusted *p* = 0.07, Fisher’s exact test).

**Table 2:** Presence of *Demodex folliculorum* and *Geotrichum candidum* on skin

Species		Observed^**a**^ (no. [%])		OR (95% CI)^**b**^	Adjusted ***p***-value^**c**^
		Presence	Absence		
*Demodex folliculorum*	Control skin	7 (15%)	39 (85%)	1	
	AD LS	33 (60%)	22 (40%)	8.2 (2.9–25.7)	< 0.003
	AD NLS	38 (69%)	17 (31%)	12.1 (4.3–38.8)	< 0.001
*Geotrichum candidum*	control skin	2 (4%)	44 (96%)	1	
	AD LS	20 (36%)	35 (64%)	12.3 (2.7–115.8)	0.04
	AD NLS	19 (35%)	36 (65%)	11.4 (2.5–107.2)	0.07

a: Species presence was defined as 10 ≥ classified reads in a sample. b: Pairwise differences between sample sites were tested using Fisher’s exact test and odds ratios were calculated using healthy control skin from non-AD individuals as the reference. c: *P*-values were corrected for mass-significance using the Bonferroni method. Adjusted *p*-values < 0.05 were considered as statistically significant. *Abbreviations*: *AD* atopic dermatitis, *LS* lesional skin, *NLS* non-lesional skin, *OR* odds ratio, *CI* confidence interval

### Distributions of *Malassezia* and *Candida*

**Fig. 3:**
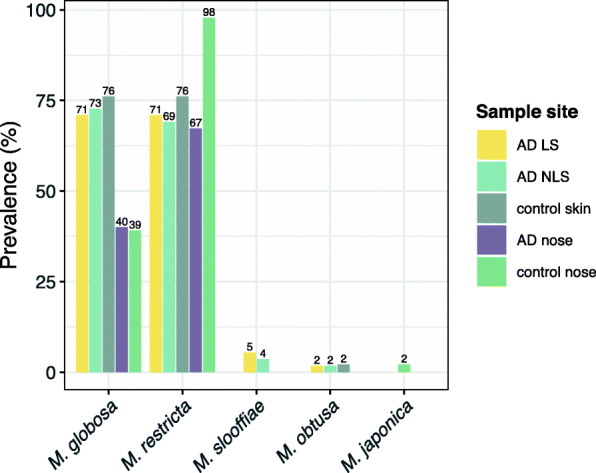
Detection frequencies of *Malassezia* spp. across sample sites. *M. globosa* was detected in 39 (71%) of the AD LS samples, 40 (73%) of AD NLS samples, 35 (76%) healthy control skin samples, 22 (40%) AD nasal samples, and in 18 (39%) healthy control nasal samples. *M. restricta* was detected in 39 (71%) AD LS samples, 38 (69%) AD NLS samples, 35 (76%) healthy control skin samples, 37 (67%) AD nasal samples, and in 45 (98%) healthy control nasal samples. *M. slooffiae* was detected in 3 (5%) and 2 (4%) of the AD LS and NLS samples respectively. *M. obtusa* was detected once among AD LS, AD NLS and healthy control skin samples. *M. japonica* was detected in a single sample taken from healthy control nares. *M. furfur* and *M. pachydermatis* were not detected in any samples. Abbreviations: AD: atopic dermatitis, LS: lesional skin, NLS: non- skin

The yeast *Malassezia* was among the most frequently detected genera at all sample sites (Additional file 1: Table S1), with *M. globosa* and *M. restricta* being the most prevalent (Fig. 3). In the anterior nares, *M. restricta* was significantly more prevalent among healthy individuals (98%) compared to AD patients (67%) (adjusted *p* = 0.03, Fisher’s exact test). Of notice, *M. furfur* was not detected in any samples.

The yeast *Candida* was more frequently identified on AD skin (LS: 64% and NLS: 65%) compared to healthy control skin (35%); however, the difference was not statistically significant. We tested if treatment with antibiotics within the last 3 months prior to sample collection was associated with the greater prevalence of *Candida* on AD skin but found no significant difference in the prevalence of *Candida* on either LS or NLS between patients, treated or not treated with antibiotics (topical and/or systemic treatment) in the past 3 months (Additional file 1: Table S2).

### Eukaryotic microbial communities in relation to clinical aspects of atopic dermatitis

There were no significant differences in either species richness or the general community composition on LS and NLS between patients with mild (SCORAD< 25, n = 18), moderate (SCORAD 25–50, *n* = 31), and severe (SCORAD> 50, n = 6) AD (Additional file 1: Fig. S6). In addition, there was no difference in either species richness or overall community composition on LS and NLS between patients with (*n* = 19) or without (*n* = 31) *FLG* mutations (Additional file 1: Fig. S7).

A hallmark of AD disease is *S. aureus* skin colonisation. Inter-species interactions and competition could very well influence *S. aureus* growth and colonisation; however, no single eukaryotic microbial species was over- or underrepresented with respect to *S. aureus* colonisation in either LS, NLS or nares. Reduced bacterial diversity, measured by Shannon-index, was not associated with changes in eukaryotic microbial richness on AD skin or in the anterior nares (Additional file 1: Fig. S8).

## Discussion

Knowledge of the eukaryotic microbial community in AD is limited [8, 9]. however, cutaneous fungi and parasites might be of importance in AD pathogenesis, as seen for the bacterial community [18]. In the present study, analysis of similarity implied that the overall eukaryotic microbial community composition on skin and in nares was significantly different between AD patients and non-AD healthy individuals. The community composition was similar on AD LS and NLS, despite clinical differences between the two sample sites. No significant association was found with either AD disease severity (SCORAD) or carriage of loss-of-function *FLG* mutations, as previously reported for the bacterial community on AD skin [5, 19]. The present study confirms that the eukaryotic microbial richness is significantly greater on AD skin compared to healthy control skin [8, 9]. This might be due to regular use of TCS among patients, as TCS treatment within 1 month prior to sample collection was associated with an increased richness on AD LS. Another possible explanation might be that antibacterial therapy alters the microbiome by reduction of bacteria thereby contributing to proliferation and changed virulence characteristics of the remaining microbes such as fungi, similar to what have previously been described for *Candida* vaginitis and *Malassezia* folliculitis after antibiotic treatment [20–22].

To some extent, the observed variation between AD and healthy control skin might be explained by a significantly higher frequency of the ectoparasite *D. folliculorum* and the fungus *G. candidum* on AD skin. *D. folliculorum* is a common skin mite that predominantly lives in sebaceous areas of the face, where it consumes sebum [23]. Though *D. folliculorum* is a commensal inhabitant of the skin, increased mite densities and penetration into the dermis have previously been associated with inflammation and skin barrier disruption [24, 25]. Also, *D. folliculorum* has been associated with the inflammatory skin disease rosacea [23, 26]. *D. folliculorum* was identified at a significantly higher prevalence on AD skin (LS as well as NLS) in this study, despite the fact that our samples primarily were collected from moist and dry skin areas. A possible explanation for this could be that AD patients regularly use moisturising lotions as part of their treatment, which might create an advantageous environment for *D. folliculorum*. This hypothesis can be supported by our observation that *D. folliculorum* was more prevalent on skin among patients reporting use of TCS, which is in alignment with findings from a study of patients with perioral dermatitis [27]. A second hypothesis could be that the skin conditions in AD itself favour *D. folliculorum* colonisation, as slightly higher colonisation frequencies have been observed among people with higher skin pH and lower skin hydration [28], as present in AD.

*G. candidum*, the other species found to be overrepresented on AD skin, is considered a common skin colonising yeast [29, 30], However, other DNA sequencing based studies on skin fungal communities, including communities on AD skin, have not reported the presence of this organism [8–11], which might be due to previously small study populations. Whether the distorted skin ecology in AD, including increased skin pH, reduced skin hydration and altered composition of free fatty acids, favours the growth of *G. candidum*, need to be further investigated.

*M. globosa* and M. *restricta* were some of the most frequently observed fungal spp. at all studied sample sites, which is in accordance with previous findings [8, 9], suggesting that these species can be considered as commensals of these anatomical areas. *Malassezia* is a lipophilic yeast, formerly thought only to reside on seborrheic skin areas (scalp, face and thorax), but after the introduction of molecular based detection methods, it has been isolated from most body sites [10]. There was no significant difference in the prevalence of *Malassezia* spp. on skin between AD patients and healthy controls, which is somewhat surprising as AD patients have a dry skin, and the genus *Malassezia* is lipid dependant. A significantly higher prevalence of *M. restricta* was found in the nares of healthy controls compared to the nares of AD patients. The explanation for this observed reduction of *M. restricta* in AD nares is not clear and needs further examination.

*Candida* is a commensal of mucosal membranes that colonises moist skin areas, and it is thus surprising that this genus tended to be more frequently detected in AD patients than healthy individuals, as patients with AD have drier skin. High prevalence of *Candida* with the potential of causing infections has previously been associated with long-term use or repeated use of antibiotics [31], however, antibiotic treatment was not associated with significant changes in *Candida* presence on AD skin in the present study.

The major strength of this study is the large cohort, which is by far the largest published to date. Furthermore, AD-diagnosis was verified by a specialised dermatologist, and all patients had active disease. This ensures high quality and clinical relevance of the presented data. A well-known limitation in the field of microbiome research is the difficulties in discriminating between colonising organisms and environmentally derived organisms. Thus, some of the identified species could be environmentally derived contaminants of the human epidermis rather than true colonisers of skin and nares, e.g. *Cladosporium* and *Aspergillus* are common spore-forming molds in both indoor and outdoor environments [17]. However, increased sensitivity in AD patients to these molds [17], together with their high prevalence found on AD and healthy control skin [8, 10], support a possible role for mold sensitivity and AD inflammation.

## Conclusion

Significant differences in the composition and richness of the eukaryotic microbial community were observed between AD patients and non-AD healthy individuals, whereas AD disease severity or filaggrin gene mutations were not associated with changes in the eukaryotic microbial community. An unexpected finding was the increased prevalence of the skin mite *D. folliculorum* on AD skin, which was associated with recent TCS treatment. Colonisation with *Demodex* spp. has been associated with folliculitis and rosacea [23, 25], and it would thus be clinically relevant to examine if *D. folliculorum* can contribute to skin inflammation in AD.

## Methods

### Study population

Adult AD patients (*n* = 58) from the outpatient clinic of the Department of Dermatology, Bispebjerg Hospital (Denmark), were included in the study in January–June 2015. Inclusion criteria were age ≥ 18 years and presence of AD according to U.K. criteria [32] as assessed by a specialised dermatologist. Exclusion criteria were pregnancy, breastfeeding and UV therapy within the last 2 months. Patients were invited to participate in the study regardless of topical of systemic treatment of AD; however, information regarding medical treatment 3 months prior to the sampling timepoint was registered (Table 1). Disease severity was assessed using the Severity Scoring of Atopic Dermatitis (SCORAD) index [33]. Blood samples were obtained for identification of the most common filaggrin gene (*FLG*) mutations among Caucasians (R501X, 2282del4, and R2447X) [34].

Non-AD healthy individuals (*n* = 46; > 18 years) were recruited among employees at Bispebjerg Hospital and Statens Serum Institut (Denmark) during the months of February and September 2016. Exclusion criteria were current or previous AD, and daily work in microbiology laboratories. The study population has previously been characterized with respect to the bacterial community composition on skin [5, 35]. All methods were carried out in accordance with relevant guidelines and regulations.

### Sample collection

Swabs (eSwabs, Copan, Italy) were taken from skin (AD LS, AD NLS, and healthy controls) and from anterior nares. Samples from AD NLS and healthy control skin were taken from the antecubital crease, except for 10 patients who had visible eczema at the site. In these cases, NLS samples were primarily taken from the volar forearm. AD LS samples were collected depending on the location of eczema, but primarily from the volar forearm and dorsal hand. Samples were stored at − 80 °C.

### DNA extraction and sequencing

DNA was extracted from samples using a MagNa Lyser instrument (Roche, Mannheim, Germany) and the FastDNA® SPIN Kit for Soil (MP Biomedicals, Santa Ana, CA, USA). The V3-V5 region of the *18S rRNA* gene was amplified in a two-step PCR using three customised primer sets [36]. Three amplicon regions were used in order to increase sequence diversity and taxonomic resolution during classification. Sequencing was carried out on a MiSeq instrument (Illumina Inc., San Diego, CA, USA) using the v2 reagent kit.

### Bioinformatics and statistics

BION v.17.10 (http://box.com/bion) was used for sequence mapping (settings described in Additional file 2: Supplemental methods) and the SILVA SSU database v.128 [37] used for taxonomic classification. *Malassezia* spp. were classified using a custom-made curated database and the DADA2 pipeline [38] (Additional file 2: Supplemental methods).

Analyses were performed in R v.3.5.1 (The R Foundation for Statistical Computing, Vienna, Austria) using the packages *phyloseq* v.1.24.2 [39], *vegan* v.2.5–3 [40], and *ggplot2* v.3.1.0 [41]. Taxa tables from the three amplicon sequence sets were merged, using the highest observed read count for each species. Sequences classified as phyla belonging to the plant kingdom or the subphylum Vertebrata were removed. Samples < 5000 reads (Additional file 1: Fig. S1) and samples from patients with incomplete sample sets were excluded.

Differences in eukaryotic microbial communities between groups were evaluated by presence/absence analysis, where species presence was defined as ≥10 reads. First, differences in overall community composition between groups were investigated using Jaccard distances and visualized with principal coordinates analysis (PCoA) plots. The degree of similarity was examined using analysis of similarity test (ANOSIM) [42] with 9999 permutations. Second, differences in species richness between groups were examined using Wilcoxon signed rank test (paired samples), Mann-Whitney U test (unpaired samples), or Kruskal Wallis test (>two unpaired groups). Species richness was compared to bacterial Shannon diversity [5] on AD skin and anterior nares using Spearman’s rank correlation test. Third, differences in the frequencies of present species between sample sites were examined using Fisher’s exact test (species present in 5–95% of samples were included in the analysis). Fisher’s exact test was also used to compare presence of pre-selected spp. between AD treatment groups. Results from each analysis were corrected for multiple testing using the Bonferroni method, and adjusted *p*-values < 0.05 were considered statistically significant (Additional file 2: Supplemental methods).

## Supplementary Information

**Additional file 1.**

**Additional file 2.**

**Additional file 3.**

**Additional file 4.**

**Additional file 5.**

## Data Availability

Amplicon sequences are available at the European Nucleotide Archive (project ID. PRJEB21789) and can be accessed at https://www.ebi.ac.uk/ena/browser/view/PRJEB21789. The presence/absence taxa tables used for analysis and a reduced sample data table (Excel files) are included as online supplemental files together with this published article (Additional files 3,4, and 5). Due to national data protection regulations regarding person-identifiable information, only a limited number of variables are included in the sample data file.
