# Supplementary material for: Identification of cutaneous fungi and mites in adult atopic dermatitis: analysis by targeted 18S rRNA amplicon sequencing
Source: BMC Microbiol. 2021 Mar 4;21:72. doi: 10.1186/s12866-021-02139-9 (PMC7934438; doi:10.1186/s12866-021-02139-9)
Supplement: Supplementary file 1 — Additional file 1. [file 12866_2021_2139_MOESM1_ESM.docx]

**Identification of cutaneous fungi and mites in adult atopic dermatitis: Analysis by targeted 18S rRNA amplicon sequencing**

Sofie Marie Edslev^1^ MSc, Paal Skytt Andersen^1,2^ PhD, Tove Agner^3^ DMSc, Ditte Marie Lindhardt Saunte, PhD^4,5^, Anna Cäcilia Ingham^1^ PhD, Thor Bech Johannesen^1^ MSc, Maja-Lisa Clausen^3^ PhD

^1^ Department of Bacteria, Parasites, and Fungi, Statens Serum Institut, Copenhagen, Denmark

^2^ Department of Veterinary and Animal Sciences, University of Copenhagen, Copenhagen, Denmark

^3^ Department of Dermatology, Bispebjerg University Hospital, Copenhagen, Denmark

^4^ Department of Dermatology, Zealand University Hopsital, Roskilde, Denmark

^5^ Department of Clinical Medicine, University of Copenhagen, Copenhagen, Denmark

**Additional file 1: Supplemental figures and tables**

**Figure S1. Sequencing depth.** Analysis is based on data derived from the three merged 18S rRNA gene amplicon datasets and was made after removing sequences classified as Mammalia or plants. **A)** A threshold of 5000 sequences in a sample was set as an inclusion threshold, which led to the loss of four samples originating from three different AD patients. B) Increasing sequencing depth was not associated with increased species richness, where species presence was defined as 10 or more reads a sample.

**Figure S2 Eukaryotic microbial community composition on atopic dermatitis skin across skin types.** Beta-diversity between skin types in **A)** LS (R = -0.06, p = 0.8, ANOSIM) and **B)** NLS (R = 0.17, p = 0.07, ANOSIM). PCoA plots and ANOSIM tests are based on Jaccard distances. Abbreviations: LS: lesional skin, NLS: non-lesional skin.

**Figure S3.** **Examination of eukaryotic microbial communities on lesional skin of atopic dermatitis patients in relation to topical corticosteroid treatment.** Patients who were treated with topical corticosteroids (TCS) in the past three months prior to sample collection were included in the TCS treatment group. **A)** Species richness on AD LS among patients treated (median species count: 30.0 (IQR: 22.5-45.8)) and not treated (20.0 (14.0-32.0)) with TCS (p = 0.05, adjusted p = 0.5). Richness was defined as the total number of species present in a sample. Boxes correspond to the 1. quartile, median, and the 3. quartile. Whiskers extend to samples with the minimum/maximum count, but no longer than 1.5 x IQR. Dots represent individual samples **B)** Beta-diversity on AD LS across treatment groups (R = 0.20, p = 0.006, adjusted p = 0.07, ANOSIM). PCoA plots and ANOSIM tests were based on Jaccard distances. Abbreviations: AD: atopic dermatitis, LS: lesional skin, TCS: Topical corticosteroid, IQR: inter-quartile-range.

**Figure S4.** **Demodex folliculorum read counts.** As species presence was defined as 10 or more classified sequences in a sample, the total number of D. folliculorum classified sequences (log10 transformed) in AD LS, AD NLS and healthy control skin samples were compared. This in order to ensure that the species presence/absence data did not imply just above or below the defined sequence threshold. The illustration shows that there indeed was a difference in the sequence count between AD skin and healthy control skin. Abbreviations: AD: atopic dermatitis, LS: lesional skin, NLS: non-lesional skin.

** Figure S5**. **Geotrichum candidum read counts.** As species presence was defined as 10 or more classified sequences in a sample, the total number of G. candidum classified sequences (log10 transformed) in AD LS, AD NLS and healthy control skin samples were compared. This in order to ensure that the species presence/absence data did not imply just above or below the defined sequence threshold. The illustration shows that there indeed was a difference in the sequence count between AD LS and healthy control skin. Abbreviations: AD: atopic dermatitis, LS: lesional skin, NLS: non-lesional skin.

**Figure S6.** **Comparison of eukaryotic microbial communities on atopic dermatitis skin across disease severity groups.** Disease severity was assessed by SCORAD and severity groups defined as: mild AD (SCORAD < 25), moderate AD (SCORAD 25-50), and severe AD (SCORAD > 50). **A)** Species richness across AD severity groups in LS (median species count (IQR). Mild AD: 34.0 (21.8-41.5), Moderate AD: 27.0 (19.5-48.0), Severe AD: 24.3 (22.8-28.8.1)) (p = 0.6, Kruskal Wallis test). **C)** Species richness across AD severity groups in NLS (Mild AD: 34.0 (28.5-43.3), Moderate AD: 30.0 (19.5-45.0), Severe AD: 27.5 (20.0-29.8)) (p = 0.2, Kruskal Wallis test). **B)** Beta-diversity in LS across AD disease severity groups visualized by PCoA (R = -0.06, p = 0.9, ANOSIM). **D)** Beta-diversity in NLS across AD disease severity groups visualized by PCoA (R = -0.06, p = 0.9, ANOSIM). For A and B: Richness was defined as the total number of species present in a sample. Boxes correspond to the 1. quartile, median, and the 3. quartile. Whiskers extend to samples with the minimum/maximum count, but no longer than 1.5 x IQR. Dots represent individual samples. For C and D: PCoA plots and ANOSIM tests were based on Jaccard distances. Abbreviations: AD: atopic dermatitis, LS: lesional skin, NLS: non-lesional skin.IQR: inter-quartile-range.

**Figure S7.** **Examination of eukaryotic microbial communities on atopic dermatitis skin in relation to filaggrin gene variants.** *FLG* mutations were defined as carriage of at least one of the following three mutations: R501X, 2282del4, and R2447X. **A)** Species richness on LS across *FLG* variants (median species count (IQR). *FLG* wildtype: 29.0 (19.5-41.5), *FLG* mutations: 25.0 (20.0-33.5)) (p = 0.8, Mann-Whitney U test). **C)** Species richness on NLS across *FLG* variants (*FLG* wildtype: 31.0 (21.0-41.0), *FLG* mutations: 29.0 (23.5-43.0)) (p = 0.7, Mann-Whitney U test). **B)** Beta-diversity in LS across filaggrin gene variants visualized by PCoA (R = -0.01, p = 0.6 ANOSIM). **D)** Beta-diversity in NLS across filaggrin gene variants visualized by PCoA (R = -0.04, p = 0.8, ANOSIM test). For A and B: Richness was defined as the total number of species present in a sample. Boxes correspond to the 1. quartile, median, and the 3. quartile. Whiskers extend to samples with the minimum/maximum count, but no longer than 1.5 x IQR. Dots represent individual samples. For C and D: PCoA plots and ANOSIM tests were based on Jaccard distances. Samples from patients with no information regarding *FLG* mutations (defined as unknown in C and D) were not included in the ANOSIM test. Abbreviations: AD: atopic dermatitis, LS: lesional skin, NLS: non-lesional skin. IQR: inter-quartile-range

**Figure S8. Bacterial Shannon diversity in relation to eukaryotic microbial richness on skin and in nares of atopic dermatitis patients.** There was no significant correlation between bacterial (16S rRNA) diversity (Shannon index) and eukaryotic microbial (18S rRNA) species richness on either **A)** LS, **B)** NLS, or **C)** anterior nares of AD patients. A linear regression line with 95% confidence interval is shown for visualization purpose. Correlation analysis was performed using the Spearman´s rank method. Abbreviations: AD: atopic dermatitis, LS: lesional skin, NLS: non-lesional skin

**Table S1. The 10 most frequent observed genera at the five sample sites**

| **Location** | **Taxa** (Phylum, Class, Order, Family, Genus) | **Count (%)** |
| --- | --- | --- |
| **AD lesional skin** (n=58) | | |
|  | Basidiomycota, Exobasidiomycetes, Malasseziales, Malasseziaceae, *Malassezia* | 49 (89%) |
|  | Ascomycota, Saccharomycetes, Saccharomycetales, Saccharomycetaceae, *Saccharomyces* | 49 (89%) |
|  | Ascomycota, Dothideomycetes, Capnodiales, Cladosporiaceae, *Cladosporium* | 45 (82%) |
|  | Ascomycota, Eurotiomycetes, Eurotiales, Trichocomaceae, *Aspergillus* | 40 (73%) |
|  | Ascomycota, Saccharomycetes, Saccharomycetales, Saccharomycetaceae, *Candida* | 35 (64%) |
|  | Arthropoda, Arachnida, Trombidiformes, Demodicidae, *Demodex* | 33 (60%) |
|  | Ascomycota, Eurotiomycetes, Eurotiales, Trichocomaceae, *Penicillium* | 33 (60%) |
|  | Ascomycota, Saccharomycetes, Saccharomycetales, Phaffomycetaceae, *Cyberlindnera* | 29 (53%) |
|  | Ascomycota;Saccharomycetes;Saccharomycetales;Saccharomycetaceae;*Debaryomyces* | 27 (49% |
|  | Basidiomycota;Tremellomycetes;Tremellales;Tremellaceae;*Cryptococcus* | 27 (49%) |
| **AD non-lesional skin** (n=58) | | |
|  | Basidiomycota, Exobasidiomycetes, Malasseziales, Malasseziaceae, *Malassezia* | 49 (89%) |
|  | Ascomycota, Saccharomycetes, Saccharomycetales, Saccharomycetaceae, *Saccharomyces* | 47 (85%) |
|  | Ascomycota, Eurotiomycetes, Eurotiales, Trichocomaceae, *Aspergillus* | 46 (84%) |
|  | Ascomycota, Dothideomycetes, Capnodiales, Cladosporiaceae, *Cladosporium* | 44 (80%) |
|  | Arthropoda, Arachnida, Trombidiformes, Demodicidae, *Demodex* | 38 (69%) |
|  | Ascomycota, Eurotiomycetes, Eurotiales, Trichocomaceae, *Penicillium* | 38 (69%) |
|  | Ascomycota, Saccharomycetes, Saccharomycetales, Saccharomycetaceae, *Candida* | 36 (65%) |
|  | Ascomycota, Saccharomycetes, Saccharomycetales, Phaffomycetaceae, *Cyberlindnera* | 35 (64%) |
|  | Basidiomycota;Tremellomycetes;Tremellales;Tremellaceae;*Cryptococcus* | 27 (49%) |
|  | Basidiomycota;Microbotryomycetes;Sporidiobolales;Sporidiobolaceae;*Rhodotorula* | 25 (46%) |
| **Healthy control skin** (n=46) | | |
|  | Basidiomycota, Exobasidiomycetes, Malasseziales, Malasseziaceae, *Malassezia* | 40 (87%) |
|  | Ascomycota, Dothideomycetes, Capnodiales, Cladosporiaceae*, Cladosporium* | 37 (80%) |
|  | Ascomycota, Saccharomycetes, Saccharomycetales, Saccharomycetaceae, *Saccharomyces* | 32 (70%) |
|  | Ascomycota, Eurotiomycetes, Eurotiales, Trichocomaceae, *Aspergillus* | 27 (59%) |
|  | Ascomycota, Eurotiomycetes, Eurotiales, Trichocomaceae, *Penicillium* | 26 (57%) |
|  | Ascomycota, Saccharomycetes, Saccharomycetales, Saccharomycetaceae*, Debaryomyces* | 23 (50%) |
|  | Ascomycota;Saccharomycetes;Saccharomycetales;Phaffomycetaceae;*Cyberlindnera* | 20 (44%) |
|  | Ascomycota;Dothideomycetes;Pleosporales;Pleosporaceae;*Alternaria* | 19 (41%) |
|  | Ascomycota;Dothideomycetes;Botryosphaeriales;Cladosporiaceae;*Verrucocladosporium* | 18 (39%) |
|  | Ascomycota;Dothideomycetes;Dothideales;Saccotheciaceae;*Aureobasidium* | 18 (39%) |
| **AD nose** (n=58) | | |
|  | Ascomycota, Dothideomycetes, Capnodiales, Cladosporiaceae, *Cladosporium* | 40 (73%) |
|  | Basidiomycota, Exobasidiomycetes, Malasseziales, Malasseziaceae, *Malassezia* | 39 (71%) |
|  | Ascomycota, Eurotiomycetes, Eurotiales, Trichocomaceae, *Aspergillus* | 34 (62%) |
|  | Arthropoda, Arachnida, Trombidiformes, Demodicidae, *Demodex* | 33 (60%) |
|  | Ascomycota, Saccharomycetes, Saccharomycetales, Saccharomycetaceae, *Saccharomyces* | 32 (58%) |
|  | Ascomycota, Eurotiomycetes, Eurotiales, Trichocomaceae, *Penicillium* | 29 (53%) |
|  | Ascomycota;Saccharomycetes;Saccharomycetales;Phaffomycetaceae;*Cyberlindnera* | 27 (49%) |
|  | Ascomycota;Saccharomycetes;Saccharomycetales;Saccharomycetaceae;*Candida* | 25 (46%) |
|  | Basidiomycota;Tremellomycetes;Tremellales;Tremellaceae;*Cryptococcus* | 17 (31%) |
|  | Ascomycota;Eurotiomycetes;Chaetothyriales;Herpotrichiellaceae;*Coniosporium* | 16 (29%) |
| **Healthy control nose** (n=46) | | |
|  | Basidiomycota, Exobasidiomycetes, Malasseziales, Malasseziaceae, *Malassezia* | 46 (100%) |
|  | Ascomycota, Dothideomycetes, Capnodiales, Cladosporiaceae, *Cladosporium* | 33 (72%) |
|  | Ascomycota, Eurotiomycetes, Eurotiales, Trichocomaceae, *Aspergillus* | 26 (57%) |
|  | Ascomycota, Saccharomycetes, Saccharomycetales, Saccharomycetaceae, *Saccharomyces* | 23 (50%) |
|  | Ascomycota;Saccharomycetes;Saccharomycetales;Saccharomycetaceae;*Candida* | 21 (46%) |
|  | Ascomycota;Eurotiomycetes;Eurotiales;Trichocomaceae;*Penicillium* | 20 (44%) |
|  | Basidiomycota;Tremellomycetes;Tremellales;Tremellaceae;*Cryptococcus* | 20 (44%) |
|  | Arthropoda;Arachnida;Trombidiformes;Demodicidae;*Demodex* | 17 (37%) |
|  | Ascomycota;Saccharomycetes;Saccharomycetales;Saccharomycetaceae;*Debaryomyces* | 17 (37%) |
|  | Basidiomycota;Microbotryomycetes;Sporidiobolales;Sporidiobolaceae;*Sporobolomyces* | 17 (37%) |

**Table S2. Presence of Candida on atopic dermatitis skin in relation to recent antibiotic treatment**

|  | **Observed^a^ in AD LS** (no. [%]) | |  |
| --- | --- | --- | --- |
|  | Presence | Absence | p-value |
| Antibiotic treatment (n = 16) | 13 (81%) | 3 (19%) | 0.1 |
| No antibiotic treatment (n = 39) | 22 (56%) | 17 (44%) |  |
|  | **Observed^a^ in AD NLS** (no. [%]) | |  |
|  | Presence | Absence | p-value |
| Antibiotic treatment (n = 16) | 10 (62%) | 6 (38%) | 0.8 |
| No antibiotic treatment (n = 39) | 26 (67%) | 13 (33%) |  |

Antibiotic treatment was defined as systemic and/or topical antibiotic treatment in the past three months. Differences in *Candida* presence on AD LS and NLS between antibiotic treated and non-treated patients were examined using Fishers exact test. a) Presence was defined as 10 ≥ classified reads in a sample. Abbreviations. AD: Atopic dermatitis, LS: lesional skin, NLS: non-lesional skin.
