# Supplementary material for: Identification of cutaneous fungi and mites in adult atopic dermatitis: analysis by targeted 18S rRNA amplicon sequencing
Source: BMC Microbiol. 2021 Mar 4;21:72. doi: 10.1186/s12866-021-02139-9 (PMC7934438; doi:10.1186/s12866-021-02139-9)
Supplement: Supplementary file 2 — Additional file 2. [file 12866_2021_2139_MOESM2_ESM.docx]

**Identification of cutaneous fungi and mites in adult atopic dermatitis: Analysis by targeted 18S rRNA amplicon sequencing**

Sofie Marie Edslev^1^ MSc, Paal Skytt Andersen^1,2^ PhD, Tove Agner^3^ DMSc, Ditte Marie Lindhardt Saunte, PhD^4,5^, Anna Cäcilia Ingham^1^ PhD, Thor Bech Johannesen^1^ MSc, Maja-Lisa Clausen^3^ PhD

^1^ Department of Bacteria, Parasites, and Fungi, Statens Serum Institut, Copenhagen, Denmark

^2^ Department of Veterinary and Animal Sciences, University of Copenhagen, Copenhagen, Denmark

^3^ Department of Dermatology, Bispebjerg University Hospital, Copenhagen, Denmark

^4^ Department of Dermatology, Zealand University Hopsital, Roskilde, Denmark

^5^ Department of Clinical Medicine, University of Copenhagen, Copenhagen, Denmark

**Additional file 2: Supplemental methods**

1. **BION settings**

Sequence clustering and species classification were made using the k-mer based tool BION. A short description of the BION settings used is given below and followed by the complete recipe.

Short description of settings:

1. A given primer set was used to extract the pairs from the raw reads with both mates present.
2. Reads were quality trimmed using a minimum quality of 98% for at least 14 of 15 bases for forward reads and 18 out of 20 for reverse reads. A minimum length of 50 was also required.
3. Read pairs were joined into one if there were overlaps of at least 20 bases with at least 75% similarity. A minimum sequence length of 300 bases and a base-call accuracy of 97%, based on the phred score, were required for passing through a sequence quality filtering step. Both overlapping and non-overlapping pairs were used.
4. Sequences were pre-clustered by 94% similarity and a minimum chimera score of 15 was used for chimera filtering.
5. Non-chimeric sequences were clustered by 97% similarity.
6. Reference matching was performed with a k-mer length of seven and a minimum oligo similarity of 60%.
7. Taxonomy tables were written for phylum, class, order, family, genus, species and sequence (strain) levels.

Complete recipe:

# -----------------------------------------------------------------------

# EXTRACTION

# -----------------------------------------------------------------------

<sequence-primer-extract>

title = Sequence extraction

trim-end-strictness = 95

pair-regex-forward = _R1_

pair-regex-reverse = _R2_

extract-reverse = no

write-failed = no

</sequence-primer-extract>

<sequence-cleaning>

# -------------------------------------------------------------------

# QUALITY TRIM

# -------------------------------------------------------------------

<sequence-trim-quality-start>

title = Start quality trim

window-length = 15

window-match = 14

minimum-quality = 98%

</sequence-trim-quality-start>

<sequence-trim-quality-end>

title = End quality trim

window-length = 20

window-match = 18

minimum-quality = 98%

</sequence-trim-quality-end>

# ---------------------------------------------------------------------

# LOW COMPLEXITY FILTER

# ---------------------------------------------------------------------

<sequence-filter>

title = Low complexity filter

pattern-string-nomatch = p1=2...2 p1 p1 p1 p1 p1 p1 p1 p1 p1

match-forward = yes

</sequence-filter>

<sequence-filter>

title = Length filtering

minimum-length = 50

</sequence-filter>

</sequence-cleaning>

# ------------------------------------------------------------------------

# JOIN PAIR MATES

# ------------------------------------------------------------------------

<sequence-join-pairs>

title = Pair mate joining

forward-filter = .F.

reverse-filter = .R.

complement-reverse = no

minimum-similarity = 75

minimum-overlap = 20

include-unjoined = yes

include-singlets = no

</sequence-join-pairs>

# -------------------------------------------------------------------------

# LENGTH AND QUALITY FILTER

# -------------------------------------------------------------------------

<sequence-cleaning>

title = Length and quality filtering

<sequence-filter>

title = Length filter

minimum-length = 300

</sequence-filter>

<sequence-filter-quality>

title = Quality filter

minimum-quality = 97

minimum-strict = 95

</sequence-filter-quality>

</sequence-cleaning>

# -------------------------------------------------------------------------

# DE-REPLICATION

# -------------------------------------------------------------------------

<sequence-dereplication>

title = Sequence uniqification

</sequence-dereplication>

# -------------------------------------------------------------------------

# CHIMERA CHECK

# -------------------------------------------------------------------------

<sequence-chimera-filter>

title = Chimera filtering

pre-clustering = 94%

maximum-chimera-score = 15

</sequence-chimera-filter>

# -------------------------------------------------------------------------

# SAMPLE CLUSTERING

# -------------------------------------------------------------------------

<sequence-clustering-samples>

title = Sample sequence clustering

oligo-word-length = 7

oligo-step-length = 1

quality-clustering-on = yes

joined-centers-only = no

quality-center-portion = 40%

minimum-base-quality = 98%

minimum-cluster-similarity = 97%

minimum-member-similarity = 70%

maximum-member-steps = 4

include-non-matches = no

</sequence-clustering-samples>

# -------------------------------------------------------------------------

# REFERENCE SIMILARITIES

# -------------------------------------------------------------------------

<sequence-similarities-simrank>

title = Making reference similarities

minimum-oligo-similarity = 60%

top-similarity-range = 8

oligo-word-length = 7

oligo-step-length = 1

</sequence-similarities-simrank>

# -------------------------------------------------------------------------

# REFERENCE PROFILING

# -------------------------------------------------------------------------

<organism-taxonomy-profiler>

title = Similarity profiling, no-split

input-step = sequence-similarities-simrank

prefer-named-taxa = yes

prefer-named-favorites = yes

noise-reduction = yes

split-close-genera = no

minimum-species-similarity = 85%

cumulative-links = no

sequence-link-ranks = domain, phylum, class, order, family, genus, species

with-debug-dumps = no

</organism-taxonomy-profiler>

<organism-profile-format>

title = Taxonomy profile tables, no-split

minimum-row-max-percent = 0%

minimum-row-value-percent = 0%

with-ambiguous-taxa = yes

with-row-sum-column = yes

normalized-column-total = 100000

</organism-profile-format>

<organism-taxonomy-profiler>

title = Similarity profiling, split

input-step = sequence-similarities-simrank

prefer-named-taxa = yes

prefer-named-favorites = yes

noise-reduction = yes

split-close-genera = yes

minimum-species-similarity = 85%

cumulative-links = no

sequence-link-ranks = domain, phylum, class, order, family, genus, species

with-debug-dumps = no

</organism-taxonomy-profiler>

<organism-profile-format>

title = Taxonomy profile tables, split

minimum-row-max-percent = 0%

minimum-row-value-percent = 0%

with-ambiguous-taxa = yes

with-row-sum-column = yes

normalized-column-total = 100000

</organism-profile-format>

</recipe>

1. **Custom taxonomic classification of *Malassezia* spp.**

We identified 16,383 unique sequences as *Malassezia* spp. by using the BION software and the SILVA SSU database v.128. However, we discovered that some of the available reference sequences of the *Malassezia* genus within the SILVA SSU database v.128 were mis-annotated. E.g. AF139485 is annotated both as *Malassezia* and *Pthirus pubis*. To achieve a reliable classification of *Malassezia* spp. at species level, we built a custom database with amplicon target sequences primarily originating from *Malassezia* spp. which were part of The CBS-KNAW culture collection (<http://www.wi.knaw.nl/Collections/DefaultInfo.aspx?Page=Home>). The custom database contained sequences from 12 different *Malassezia* spp. (Table 1); however, the reference sequences of four of the species (*M. dermatis*, *M. sympodialis*, *M. caprae*, and *M. equina)* were identical (Table 2) and these species could therefore not be discriminated in the present study.

We mapped all unique *Malassezia* spp. sequences against our database using the *assignTaxonomy()* function in the R package dada2, which uses the RDP naïve Bayesian classifier method. 15,774 out of 16,383 sequences could thereby be classified at species level. The sequence count table was subsequently agglomerated at species level for downstream analyses.

**Table 1.** Custom database Malassezia spp. list

| **Species** | **CBS no.** | **Genbank accession no.** |
| --- | --- | --- |
| *M. globosa* | 7966 | AAYY01000016.1 |
| *M. restricta* | 7877 | AAXK01002636.1 |
| *M. furfur* | 1817 | KF706457.1 |
| *M. obtusa* | 7968 | KF706455.1 |
| *M. Slooffiae* | 7956 | KF706459.1 |
| *M. japonica* | 9431 | KF706458.1 |
| *M. dermatis* | 9169 | KF706452.1 |
| *M. sympodialis* | Non CBS isolate | EU192369.1 |
| *M. nana* | 9558 | KF706453.1 |
| *M. pachydermitis* | Non CBS isolate | EU192366.1 |
| *M. caprae* | 10434 | NG_063001.1 |
| *M. equina* | 9969 | KF706454.1 |

Table 2. Pairwise comparison of amplicon target sequences in the custom database of Malassezia spp.

|  |  | 1 | 2 | 3 | 4 | 5 | 6 | 7 | 8 | 9 | 10 | 11 | 12 |
| --- | --- | --- | --- | --- | --- | --- | --- | --- | --- | --- | --- | --- | --- |
| *M. dermatis* | 1 |  |  |  |  |  |  |  |  |  |  |  |  |
| *M. sympodialis* | 2 | 0 |  |  |  |  |  |  |  |  |  |  |  |
| *M. caprae* | 3 | 0 | 0 |  |  |  |  |  |  |  |  |  |  |
| *M. equina* | 4 | 0 | 0 | 0 |  |  |  |  |  |  |  |  |  |
| *M. nana* | 5 | 3 | 3 | 3 | 3 |  |  |  |  |  |  |  |  |
| *M. globosa* | 6 | 8 | 8 | 8 | 8 | 7 |  |  |  |  |  |  |  |
| *M. restricta* | 7 | 9 | 9 | 9 | 9 | 10 | 5 |  |  |  |  |  |  |
| *M. pachydermitis* | 8 | 12 | 12 | 12 | 12 | 15 | 13 | 14 |  |  |  |  |  |
| *M. furfur* | 9 | 32 | 32 | 32 | 32 | 32 | 31 | 34 | 31 |  |  |  |  |
| *M. obtusa* | 10 | 30 | 30 | 30 | 30 | 30 | 29 | 32 | 29 | 5 |  |  |  |
| *M. japonica* | 11 | 26 | 26 | 26 | 26 | 26 | 23 | 26 | 25 | 17 | 18 |  |  |
| *M. slooffiae* | 12 | 38 | 38 | 38 | 38 | 38 | 36 | 37 | 40 | 30 | 27 | 27 |  |

1. **P-value adjustments using Bonferroni correction**

P-values were adjusted for a subset of analyses at a time:

1. ANOSIM tests = 9 tests included in adjustment:

[comparison of sample sites: LS vs NLS]

[comparison of sample sites: LS vs control skin]

[comparison of sample sites: NLS vs control skin]

[comparison of sample sites: AD vs control nares]

[comparison of TCS groups, AD LS]

[comparison of SCORAD groups, AD LS]

[comparison of SCORAD groups, AD NLS]

[comparison of *FLG* groups, AD LS]

[comparison of *FLG* groups, AD NLS]

1. Richness tests = 9 tests included in adjustment:

[comparison of sample sites: LS vs NLS]

[comparison of sample sites: LS vs control skin]

[comparison of sample sites: NLS vs control skin]

[comparison of sample sites: AD vs control nares]

[comparison of TCS groups, AD LS]

[comparison of SCORAD groups, AD LS]

[comparison of SCORAD groups, AD NLS]

[comparison of *FLG* groups, AD LS]

[comparison of *FLG* groups, AD NLS]

1. Fisher’s tests; comparison of species prevalences between AD LS and NLS sample sites = 118 tests included in adjustment.
2. Fisher’s tests; comparison of species prevalences between AD LS and control skin = 118 tests included in adjustment.
3. Fishers tests; comparison of species prevalences between AD NLS and control skin = 118 tests included in adjustment.
4. Fishers tests, comparison of species prevalences between AD and control nares = 118 tests included in adjustment.
5. Fisher’s tests; comparison of selected species prevalences between AD treatments groups = 3 tests included in the adjustment:

[*Dermodex folliculorum* presence, TCS treatment, AD LS]

[*Canida* presence, antibiotic treatment, AD LS]

[*Canida* presence, antibiotic treatment, AD NLS]

1. Spearman’s rank correlation tests, comparison of bacterial shannon diversity and eukaryotic microbial species richness = 3 tests included in adjustment.
